# The Impact of Different *MEFV* Genotypes on Clinical Phenotype of Patients with Familial Mediterranean Fever: Special Emphasis on Joint Involvement

**DOI:** 10.1007/s00431-024-05716-y

**Published:** 2024-08-07

**Authors:** Esma Aslan, Nergis Akay, Umit Gul, Elif Kilic Konte, Aybuke Gunalp, Fatih Haslak, Amra Adrovic, Kenan Barut, Mehmet Yildiz, Sezgin Sahin, Ozgur Kasapcopur

**Affiliations:** Department of Pediatric Rheumatology, Cerrahpasa Medical School, Istanbul University-Cerrahpasa, Istanbul, Türkiye

**Keywords:** Arthritis, Familial Mediterranean fever, Genotypes, HLA-B27, Enthesitis-related arthritis

## Abstract

Familial Mediterranean Fever (FMF) is the most common monogenic autoinflammatory disease worldwide. In this retrospective cohort study, we aimed to assess the effects of various *MEFV* genotypes on the clinical characteristics of the patients, with a special focus on the joint involvement. In total, 782 patients with FMF were categorized into 3 groups according to the *MEFV* mutation; Group 1: Patients homozygous for M694V; Group 2: Patients carrying other pathogenic *MEFV* variants in exon 10 in homozygous or compound heterozygous states; and Group 3: FMF patients with other variants or without mutations. Clinical and demographic findings were compared between groups. Among the 782 FMF patients, total frequency of arthritis was 237 (30.3%): 207 (26.4%) were acute monoarthritis and 67 (8.5%) were chronic arthritis. Both the frequency of arthritis (acute and/or chronic) (40.4% vs. 24.8% vs. 26.7%; p:0.001) and acute monoarthritis (35.4% vs. 20% vs. 23.7%; p:0.001) were significantly higher in Group 1 than in the other groups. FMF patients with chronic arthritis showed a distinct juvenile idiopathic arthritis (JIA) distribution pattern with a more frequent enthesitis-related arthritis (ERA) subtype (43.2%). HLA-B27 was positive in 24% of the ERA patients.

*Conclusion*: Homozygous M694V mutation is associated with a more frequent and longer acute monoarthritis comparing to other *MEFV* genotypes. In addition, the risk of chronic arthritis seems not related to the *MEFV* mutations. However, FMF patients with chronic arthritis show a distinct ILAR JIA distribution pattern with a more frequent ERA and undifferentiated arthritis subtype.

**Table:** 

**What is known:**
*• Homozygous M694V mutation is associated with a more frequent and longer acute monoarthritis*
**What is new:**
*• FMF patients with chronic arthritis show a distinct ILAR JIA distribution pattern with a more frequent ERA subtype*
*• ERA patients with negative HLA-B27 antigen should also be assessed for polyserositis episodes of FMF, especially in countries with high FMF carrier frequency*

## Introduction

Familial Mediterranean fever (FMF) is the most common monogenic autoinflammatory disease worldwide [1]. It has a strong ethnic distribution in Mediterranean and Middle Eastern societies, such as Turks, Armenians, Jews, and Arabs [2]. It is characterized by fever and self-limiting attacks of polyserositis that occur at irregular intervals, usually lasting 12 to 72 h [3]. The *MEFV* (Mediterranean fever) gene, the causative gene of the disease, is located on the short arm of chromosome 16 and consists of 10 exons [4]. More than 350 sequence analyses associated with FMF have been identified in the *MEFV* gene. Although pathogenic or likely pathogenic variants are mostly located in exon 10, many other mutations in exons 2, 3, and 5 are also associated with FMF [5–7]. The type of mutations in *MEFV* gene impact clinical findings and disease phenotype [8, 9].

Familial Mediterranean fever is characterized by inflammatory attacks lasting between 6 and 72 h. During these inflammatory episodes, fever, generalized abdominal pain, chest pain, erysipelas-like rash are characteristic findings of the disease. Joint involvement is one of the common and important findings of FMF and can sometimes be the first sign of the disease in children [5, 8, 10]. The prevalence of arthritis among individuals with FMF varies across different ethnic groups. It appears that the prevalence is lower in Arabs, Turks, and Armenians when compared to Jews [11] [12]. Patients with FMF typically experience temporary monoarthritis affecting the large joints of their lower extremities. This condition usually lasts for 7 to 10 days, which is longer than other symptoms. It is important to note that the arthritis seen in FMF is generally non-erosive and does not lead to long-term sequale. However, in rare cases, up to 2–5% of patients may develop chronic arthritis in the hips or knees [5]. Additionally, there are increasing number of cases where sacroiliitis accompanies FMF in the literature [13].

The primary goal of this retrospective cohort study was to evaluate the clinical features of patients, identify the prevalence and characteristics of arthritis in children with FMF, and investigate the influence of MEFV gene variants on their clinical features in a substantial pediatric FMF population.

## Material and methods

### Study population and definitions

The present retrospective cohort study enrolled patients who had been diagnosed with FMF before the age of 18, Istanbul Unıversity-Cerrahpasa Department of paediatric rheumatology between September 2022 and May 2023. The inclusion criteria were that the patients met the Turkish paediatric FMF criteria, had a follow-up period of at least 6 months, and the patient/legal representative agreed to participate in the study. The Turkish paediatric FMF criteria set includes the following items: fever (at least three attacks, lasting 6–72 h), abdominal pain (at least three attacks, lasting 6–72 h), chest pain (at least three attacks, lasting 6–72 h), arthritis (at least three attacks lasting 6–72 h, oligoarthritis) and family history. At least two of these five criteria must be present for the diagnosis of FMF [7]. Patients who did not meet the Turkish pediatric FMF criteria, patients with a follow-up period of less than 6 months, patients with fundamental deficiencies in disease-related data, and patients who did not volunteer to participate in the study were excluded from the study. The study was initially planned to include 850 patients. However, seventeen patients did not meet the Turkish criteria for pediatric FMF, forty-three patients had a follow-up period of less than six months, seven patients had essential deficiencies in disease-related data, and one patient did not volunteer to participate in the study; therefore, they were excluded. Consequently, a total of 782 patients were included in the study. Demographic, clinical, genetic, and laboratory data of the patients were obtained from the patient records and these data were confirmed by the patients/parents during the outpatient clinic examination.

Patients with arthritis were allocated into two categories: those with acute monoarthritis and those with chronic arthritis*.* Chronic arthritis was defined as any arthritic condition that persisted for more than six weeks. Patients diagnosed with chronic arthritis and meeting the International Association of Rheumatology Associations (ILAR) classification criteria were subsequently categorized into one of seven subtypes of Juvenile Idiopathic Arthritis (JIA) according to the ILAR definitions: systemic JIA, oligo articular JIA, rheumatoid factor positive polyarticular JIA, rheumatoid factor negative polyarticular JIA, enthesitis-related arthritis (ERA), psoriatic arthritis, and undifferentiated JIA [14].

Colchicine resistance is defined as having at least one attack per month over the last 3 months despite the highest tolerated dose in patients who have good colchicine compliance [15]. According to recommendations, the standard colchicine dose is 1.2 mg/m^2^/day. The colchicine dose for children under 5 years of age is 0.5 mg/day; 0.5–1 mg/day for children between the ages of 5 and 10, and 1.5 mg/day for children older than 10 [16]. In case of colchicine resistance, colchicine is first increased to the highest tolerated dose. If the attacks are not controlled, a different brand of colchicine is tried. Anti- interleukin-1 (IL-1) treatment is approved in patients whose attacks continue despite all these interventions.

### Genetic analysis of MEFV gene

All patients were analysed for sequence variants in exons 2, 3, 5, and 10 of the *MEFV* gene. DNA isolation from blood samples was performed according to standard procedures. The test uses polymerase chain reaction (PCR) and reverse hybridization (FMF StripAssay, ViennaLab). The study group was divided into 3 groups according to the pathogenicity of *MEFV* mutation. Group 1: Patients homozygous for M694V; Group 2: Patients carrying other pathogenic *MEFV* variants (M694I, M680I, V726A) in exon 10 in homozygous or compound heterozygous states; and Group 3: FMF patients with other variants (whether homozygous or heterozygous) or without mutations. All these 3 groups were compared in terms of demographic data, clinical features, and joint involvement [2, 17].

### Ethical approval

The Istanbul University-Cerrahpasa Institutional Review Board approved this study. The approval date and protocol number are 07.12.2022–558367. Written informed consent was received from the legal representatives of patients. The study was performed according to the principles of the Declaration of Helsinki.

### Statistical analysis

Statistical analyses were performed using the IBM SPSS 20.0 program. The Kolmogorov–Smirnov test was used to analyse the distributions of variables. Continuous variables with a normal distribution are given as mean ± standard deviation (SD), and those that do not follow a normal distribution are given as median (minimum–maximum). The chi-square test was applied to compare categorical variables in patients categorized according to the *MEFV* genotype. For continuous variables that were not normally distributed, the Kruskal–Wallis test was used. Statistical significance is defined as *p*-value < 0.05.

## Results

### Demographic and clinical features

A total of 782 FMF patients were included in the study. Three hundred eighty-four of them (49.1%) were female. The average age of the patients at the time of the study was 14.2 ± 4.4 years. The median age ​​of disease onset and diagnosis were 3 (0–16) years and 6 (1–17) years, respectively. Abdominal pain (82.9%), fever (79.2%), and arthralgia (63.4%) were the most frequently reported symptoms by patients. Acute monoarthritis was the fourth most common manifestation, which was observed in 207 (26.5%) patients. All patients received colchicine treatment. Of the 52 (6.6%) patients who had colchicine resistance, 47 (6%) patients were on IL-1 inhibitors and 5 (0.6%) patients were in the process of getting approval from the health authorities. Consanguineous marriage was observed in 219 (28%) patients. Two (0.25%) patients had renal amyloidosis (Table 1).

**Table 1:** Demographic data, symptoms and clinical presentations of patients in our cohort study

	n (%) or mean ± SD or median (min -max)
Total number of patients	782
Gender (female)	384 (49.1%)
Age at last visits (years)	14.2 ± 4.4
Age of symptom onset (years)	3 (0–16)
Age of disease diagnosis (years)	6 (1–17)
Fever	619 (79.2%)
Abdominal pain	648 (82.9%)
Chest pain	164 (21%)
Arthritis	237 (30.3%)
-Acute monoarthritis	207 (26.5%)
-Chronic Arthritis	67 (8.5%)
Arthralgia	496 (63.4%)
Erysipelas-like erythema	30 (3.8%)
Prolonged febrile myalgia	8 (1%)
Renal amyloidosis	2 (0.2%)
Colchicine resistance	52 (6.6%)
The dose of colchicine (mg/day)	1.47 ± 0.6
Biological agent requirement Canakinumab Adalimumab Etanercept Anakinra Tocilizumab Certolizumab	91 (11.6%) 40 (5.1%) 27 (3.4%) 15 (1.9%) 7 (0.9%) 1 (0.1%) 1 (0.1%)

***SD*** standart deviation ***min.*** minimum, ***max.*** maximum

One hundred eighty-four of our patients (23.5%) had at least one comorbid disease. The most common comorbid disease was JIA (*n* = 67, 8.5%), followed by periodic fever, aphthous stomatitis, pharyngitis and adenitis (PFAPA) syndrome (*n* = 17, 2.1%), allergic asthma (*n* = 14, 1.8%) and immunoglobin A (IgA) vasculitis (*n* = 8, 1%). Among FMF patients with comorbid disease, 43 patients (23.4%) anti-tumour necrosis factor (TNF) therapy, 36 patients (19.5%) methotrexate, 3 patients (1.6%) azathioprine, 2 patients (1.0%) mycophenolate mofetil, and 2 patients (1.0%) anti-interleukin 6 (IL-6) treatment.

### MEFV gene variants of the study population

The Mediterranean fever gene variant distribution in our study was as follows: M694V allele in 518 (66.2%) patients, R202Q allele in 195 (24.9%), M680I allele in 111 (14.2%), V726A allele in 87 (11.1%) and E148Q allele in 71 (9%) patients. The most common *MEFV* genotype was M694V homozygous mutation (*n* = 223, 28.5%), followed by M694V heterozygous mutation (*n* = 124, 15.8%). No mutation was detected in 55 (7%) patients (Table 2). Among 184 patients with comorbidities, the most common genotype was M694V homozygous mutation (12%), and the most common allele was M694V allele (15%).

**Table 2:** Distribution of different *MEFV* variants in the entire study group

Mutations	n (%)
M694V Homozygous M694V Heterozygous R202Q Homozygous R202Q Heterozygous M694V/M680I Compound Heterozygous M694V/R202Q Compound Heterozygous M694V/V726A Compound Heterozygous E148Q Heterozygous V726A Heterozygous M680I Heterozygous M680I Homozygous M694V/E148Q Compound Heterozygous M694V/R761H Compound Heterozygous No variant detected Others	223 (%28.5) 124 (%15.8) 81 (%10.3) 64 (%8.1) 54 (%6.9) 44 (%5.6) 40 (%5.1) 40 (%5.1) 28 (%3.5) 24 (%3) 19 (%2.4) 17 (%2.1) 12 (%1.5) 55 (%7) 82 (%10.4)

### Comparisons according to the genotypes of the patients

Based on genotypes of the patients; study cohort were divided into 3 groups: Patients homozygous for M694V (Group 1), patients carrying other pathogenic *MEFV* variants (M694I, M680I, V726A) in exon 10 either in a homozygous or compound heterozygous state (Group 2) and FMF patients with other variants (homozygous, heterozygous or compound heterozygous) or without any mutation (Group 3). There were 223 patients in Group 1, 125 in Group 2, and 434 in Group 3. The age at disease onset (2 years vs. 3 years vs. 4 years; *p* < 0.001) and the age at diagnosis (4 years vs. 5 years vs. 6 years; *p* < 0.001) in Group 1 were earlier than the other groups. The frequency of arthritis (35.4% vs. 20% vs. 23.7%; p:0.001) and erysipelas-like erythema (ELE) (8.5% vs. 2.4% vs. 1.8%; *p* < 0.001) were also significantly higher in Group 1 than in the other groups. Chest pain in Group 2 (31.2%) was more pronounced comparing to Group 1 (25.6%) and Group 3 (15.7%) (*p* < 0.001). The average number of FMF episodes in the last 6 months (1 vs. 0 vs. 0; *p* < 0.001) was higher in Group 1 than in the other groups. The mean colchicine dose in all study cohort was 1.47 ± 0.6 mg/day. Additionally, the average colchicine dose (1.64 ± 0.5 mg vs. 1.5 ± 0.6 mg vs. 1.38 ± 0.64 mg; *p* < 0.001), the frequency of colchicine resistance (16.6% vs. 6.4% vs. 1.6%; *p* < 0.001), the need for a different colchicine brand (8.5% vs. 1.6% vs. 0.9%; *p* < 0.001) and the use of anti-IL1 therapy (14.3% vs. 6.4% vs. 1.6%; *p* < 0.001) was higher in Group 1 than in Group 2 and Group 3. No significant difference was detected between the groups in terms of comorbidities, consanguineous marriages, and the presence of chronic arthritis. There were 2 patients with renal amyloidosis; one was harbouring the M694V homozygous (Group 1) mutation and the other patient compound heterozygous M680I/V726A (Group 2) mutation.

### Joint involvement

The total frequency of arthritis in our cohort was 237 (30.3%). Of these, 207 (26.4%) were acute monoarthritis and 67 (8.5%) were chronic arthritis. Among the 237 patients with arthritis, 37 developed both acute and chronic arthritis in the course of the disease. Among the patients with chronic arthritis, the most frequent 3 genotypes in descending order were M694V homozygous mutation (n = 22, 32.8%), M694V/M680I compound heterozygous mutation (*n* = 9, 13.4%) and M694V heterozygous mutation (*n* = 9, 13.4%). Other less frequent mutations were as follows: M694V/R202Q compound heterozygous in 5 patients (7.5%), M694V/V726A compound heterozygous in 4 patients (5.9%), E148Q heterozygous in 3 patients (4.5%), R202Q heterozygous in two patients (3%), M694V/E148Q compound heterozygous in two patients (3%), M680I/R202Q compound heterozygous in one patient (1.5%), M680I heterozygous in one patient (1.5%), M680I homozygous in one patient (1.5%), M680I/E148Q compound heterozygous in one patient (1.5%), V726A homozygous in one patient (1.5%), V726A/R761H compound heterozygous in one patient (1.5%), V726A heterozygous in two patients (3%), and R202Q homozygous in one patient (1.5%). No mutations were detected in two patients (3%). Additionally, the most common alleles in this group are M694V allele (*n* = 51, 6.5%), R202Q allele (*n* = 22, 2.8%), and M680I allele (*n* = 12, 1.5%), respectively. The distribution of JIA subgroups in patients with chronic arthritis was as follows: enthesitis-related arthritis (ERA) in 29 patients (43.2%), oligoarticular JIA (oJIA) in 13 (19.4%), polyarticular JIA (polyJIA) in 8 (11.9%), systemic JIA (sJIA) in 2 (3%), juvenile psoriatic arthritis (jPsA) in 1 (1.5%), and undifferentiated chronic arthritis in 14 patients (20.9%). Of the 25/29 ERA patients who have been analysed for the presence of HLA-B27 antigen, 24% (*n* = 6) was positive for HLA-B27 antigen.

All 3 groups were compared in terms of the frequency of arthritis (acute and/or chronic), acute monoarthritis and chronic arthritis. Both the frequency of arthritis (40.4% vs. 24.8% vs. 26.7%; p:0.001) and acute monoarthritis (35.4% vs. 20% vs. 23.7%; p:0.001) were found to be significantly higher in Group 1 than in the other groups. The average duration of acute monoarthritis (2 days vs. 1 day vs. 1 day; p:0.001) was longer in patients with M6964V homozygous mutation. The rate of arthralgia and chronic arthritis did not differ between groups (Table 3).

**Table 3:** Comparison of the characteristics of familial Mediterranean fever-related arthritis according to genotype

	Total n (%)	Group 1* n (%)	Group 2* n (%)	Group 3* n (%)	p value
Patients	782 (100%)	223 (100%)	125 (100%)	434 (100%)	
Patients with arthritis	237 (30.3%)	91 (40.8%)	31 (24.8%)	115 (26.5%)	*p* = **0.001**
Acute monoarthritis	207 (26.5%)	79 (35.4%)	25 (20%)	103 (23.7%)	*p* = **0.001**
Chronic Arthritis	67 (8.5%)	22 (9.8%)	14 (11.2%)	31 (7.1%)	0.35
ERA	29 (43.2%)	12 (54.5%)	3 (20%)	14 (46.7%)	0.27
oJIA	13 (19.4%)	4 (18.2%)	3 (20%)	6 (20%)	0.72
PolyJIA	8 (11.9%)	1 (4.5%)	4 (26.7%)	3 (10%)	*p*< **0.05**
sJIA	2 (3%)	1 (4.5%)	-	1 (3.3%)	0.72
JPsA	1 (1.5%)	-	-	1 (3.3%)	0.67
uJIA	14 (20.9%)	4 (18.2%)	4 (26.7%)	6 (20%)	0.40
Acute monoarthritis attack duration (day) (median, min–max)	2 (0–30)	2 (0–30)	1 (0–7)	1 (0–14)	*p* < **0.05**

***ERA*** enthesitis-related arthritis, ***oJIA*** oligoarticular juvenile idiopathic arthritis, ***PolyJIA*** polyarticular juvenile idiopathic arthritis, ***sJIA*** systemic juvenile idiopathic arthritis, ***JPsA*** juvenile psoriatic arthritis, ***uJIA*** undifferentiated JIA, ***SD*** standart deviation ***min.*** minimum, ***max.*** maximum

^*^Group 1: Patients homozygous for M694V ***Group 2:*** patients carrying other pathogenic *MEFV* variants (M694I, M680I, V726A) in exon 10 either in a homozygous or compound heterozygous state ***Group 3:*** Patients with other variants (homozygous, heterozygous or compound heterozygous) or without any mutation

## Discussion

Our study investigated the impact of genotype on the demographic and clinical characteristics in a large paediatric FMF cohort. In addition, we aimed to particularly focus on the association of 3 different genotype groups with joint involvement. Familial Mediterranean fever patients homozygous for M694V are likely to develop acute monoarthritis, which also lasts longer comparing to other genotypes.

Arthritis, which is the presenting manifestation in a sort of FMF patients, is not uncommon in FMF [5, 8]. In previously published studies, the frequency of arthritis varies between 18.5% and 43.2%, and the frequency of arthralgia varies between 31% and 63.7% [1, 18–23]. Consistent with these papers, a similar rate of arthritis (26.5%) and arthralgia (63.4%) was observed in our patient cohort.

The presence of the M694V allele, especially the M694V homozygous mutation is closely associated with musculoskeletal manifestations. Besides, several other manifestations including arthritis, arthralgia and exertional leg pain are also more common in M694V homozygous patients [18, 23–26]. Likewise, the frequency of arthritis and acute monoarthritis were higher, and the duration of acute monoarthritis was longer in the M694V homozygous group compared to the other two groups in our cohort. However, there was no difference between genotype groups in the frequency of chronic arthritis.

The frequency of several diseases increases in FMF patients, possibly due to the impaired imbalance between the pro- and anti-inflammatory pathways of the innate immune system [13, 27–30]. Additionally, many studies have reported that the pathogenic alleles in exon 10, especially M694V, increase the frequency of comorbid diseases [27, 28, 31]. Besides, individuals who may also have other susceptibility factors (e.g., FMF) for JIA are at a higher risk of developing chronic arthritis [32]. Data on the long-term outcomes of chronic arthritis in patients with FMF are limited. In a Syrian study, of the 71 FMF patients with arthritis, two patients (3%) developed joint deformity and required hip replacement surgery [25].

In our cohort, among the 184 patients with comorbidities, the most common disease was JIA, with a frequency of 36.4%. However, no significant difference was detected in the frequency of comorbidities between M694V homozygous group and the other groups. The most frequent JIA subtypes in our FMF patients with chronic arthritis were ERA (43.2%), undifferentiated JIA (20.9%) and oJIA (19.4%). In a previous study conducted on the frequency of JIA subgroups in our country, the most common subtypes were oligoarticular JIA (38.8%) and ERA (23.2%), followed by polyarthritis (17.8%), systemic arthritis (12.2%), JPsA (5.2%), and undifferentiated arthritis (2.8%) [33]. Compared to JIA cohort without FMF, our FMF cohort displayed a distinct JIA subtype distribution with a more frequent ERA and undifferentiated arthritis subtypes. Similar to our study, recent studies have also reported that the incidence of ERA has increased in patients with FMF [13, 34, 35]. Higher rates of sacroiliitis, which has been reported as the most frequent chronic joint involvement in FMF patients, may explain the predominance of ERA subtype in our FMF cohort. Besides, our FMF plus ERA patients have exhibited lower rates of HLA-B27 positivity comparing to a previously multicentre JIA study conducted in our country (24.0% vs. 51.7%) [33].

In previous reports, M694V homozygous mutation is associated with a more severe clinical phenotype with an earlier disease onset and lower response to colchicine treatment [28, 36, 37]. Also, in FMF patients with arthritis, the required colchicine dose in the treatment and the rate of colchicine unresponsiveness were higher than the average FMF population [38]. In our study, consistent with the literature, earlier age at disease onset and diagnosis, and higher-doses of colchicine requirement were observed in the M694V homozygous group compared to the other two groups. The treatment algorithm for FMF patients with arthritis should be based on the EULAR recommendations, which propose intra-articular steroid injections and the use of synthetic or biological DMARDs [39].

The most important limitation of our study is its retrospective design and absence of data regarding which joints are affected during the course of the disease. Our strengths are that the *MEFV* gene analysis was performed in all patients of our cohort, longer follow-up period and detailed assessment of joint involvement patterns.

In conclusion, homozygous M694V mutation is associated with a more frequent and longer acute monoarthritis comparing to other *MEFV* genotypes. In addition, the risk of chronic arthritis seems not related to the *MEFV* mutations. However, FMF patients with chronic arthritis show a distinct ILAR JIA distribution pattern with a more frequent ERA and undifferentiated arthritis subtype.

## Data Availability

No datasets were generated or analysed during the current study.

## Abbreviations

ELE: Erysipelas-like erythema
ERA: Eenthesitis-related arthritis
FMF: Familial Mediterranean Fever
HLAB-27: Human Leukocyte Antigen B-27
IgA: Immunoglobin A
IL: Interleukin
ILAR: International Association of Rheumatology Associations
JIA: Juvenile Idiopathic Arthritis
JPsA: Juvenile psoriatic arthritis
Max: Maximum
MEFV: Mediterranean fever
Min: Minimum
oJIA: Oligoarticular JIA
PCR: Polymerase chain reaction
PFAPA: Periodic fever, aphthous stomatitis, pharyngitis and adenitis
PolyJIA: Polyarticular JIA
SD: Standard deviation
sJIA: Systemic JIA
TNF: Tumour necrosis factor

## Declarations

### The patient and public involvement statement

Patients or the public were not involved in the design, or conduct, or reporting, or dissemination plans of our research.

### Data sharing statement

All data relevant to the study are included in the article.

### Previous presentation

The data from this study were presented as an oral presentation at the Pediatric Rheumatology European Society (PReS) Congress-the Young Investigators Meeting; on September 28 2023, in Rotterdam, Netherlands (Abstract; ABS-1774).
